# A Common Language for Post-Transplant Anastomotic Biliary Strictures: Standardized Endoscopic Outcome Definitions and a Failure-Mode Roadmap

**DOI:** 10.3390/medicina62091773

**Published:** 2026-09-15

**Authors:** Daniele Balducci, Michele Montori, Marco Marzioni, Giuseppe Tarantino, Antonio Benedetti, Gianluca Svegliati-Baroni, Emanuele Bendia, Enrico Palmeri, Matteo Ghisa, Luca Maroni

**Affiliations:** 1 Clinic of Gastroenterology, Hepatology, and Emergency Digestive Endoscopy, Università Politecnica Delle Marche, 60126 Ancona, Italy; 2 Liver Injury and Transplant Unit, Gastroenterology and Transplant Department, Polytechnic University of Marche, 60126 Ancona, Italy; 3 Gastroenterology and Transplant Department, Digestive Endoscopy and Chronic Inflammatory Bowel Diseases, Ospedali Riuniti di Ancona, 60126 Ancona, Italy; 4 Gastrointestinal Endoscopy Unit, Gastroenterology Department, Padova University Hospital, 35128 Padua, Italy

**Keywords:** liver transplantation, post-liver transplant biliary stricture, anastomotic biliary stricture, endoscopic retrograde cholangiopancreatography, biliary stenting, fully covered self-expandable metal stent, multiple plastic stents, refractory biliary stricture, non-anastomotic stricture, cholangioscopy

## Abstract

*Background and Objectives***:** Anastomotic biliary stricture (AS) is the most frequent biliary complication after duct-to-duct liver transplantation (LT) and a leading indication for post-transplant endoscopic retrograde cholangiopancreatography (ERCP). Because inconsistent outcome definitions hamper cross-study comparisons, we also propose standardized outcome definitions and a minimum reporting dataset. *Materials and Methods:* This narrative review covers pathogenesis and risk factors, diagnosis and timing, balloon dilation and stenting, the management of treatment failure, procedure-related safety, and graft outcomes. *Results:* ERCP is the established first-line treatment and achieves durable resolution in most patients. Randomized evidence shows comparable resolution with multiple plastic stents (MPS) and fully covered self-expandable metal stents (FCSEMS), the latter reducing procedures and treatment duration at the cost of migration and possible recurrence. Evidence is weaker for recurrent or refractory strictures, sequential stent addition versus complete exchange, and Roux-en-Y anatomy or a completely obstructed anastomosis. We define technical success, treatment success, recurrence, refractory disease, and durable stent-free resolution, and organize advanced management as a roadmap matching four modes of endoscopic failure to salvage techniques. *Conclusions:* Endoscopic therapy is central to graft-preserving management of post-transplant AS; adopting these harmonized definitions should make outcomes comparable and rescue algorithms evidence-based.

## 1. Introduction

Biliary complications remain the “Achilles’ heel” of liver transplantation, and among them, anastomotic stricture (AS) is the most common, the most amenable to endoscopic therapy, and the one with the most favorable prognosis when successfully treated [1,2]. AS is a focal, fibrotic narrowing at the bilio-biliary (duct-to-duct) or bilio-enteric anastomosis. It must be distinguished, conceptually and therapeutically, from non-anastomotic strictures (NAS), which are typically multifocal, intrahepatic, ischemic or immune-mediated, less responsive to endoscopy, and associated with a worse prognosis [1,3].

The clinical importance of AS lies in its frequency and in the availability of an effective, organ-preserving, minimally invasive treatment. In recipients with duct-to-duct reconstruction, ERCP has become the established first-line modality, displacing surgical revision for the majority of patients [4,5]. Yet several practical questions continue to divide endoscopists: which stent to use, how aggressively to escalate treatment, how to define success and failure, and whether durable endoscopic resolution actually translates into better graft and patient survival.

**Figure 1:**
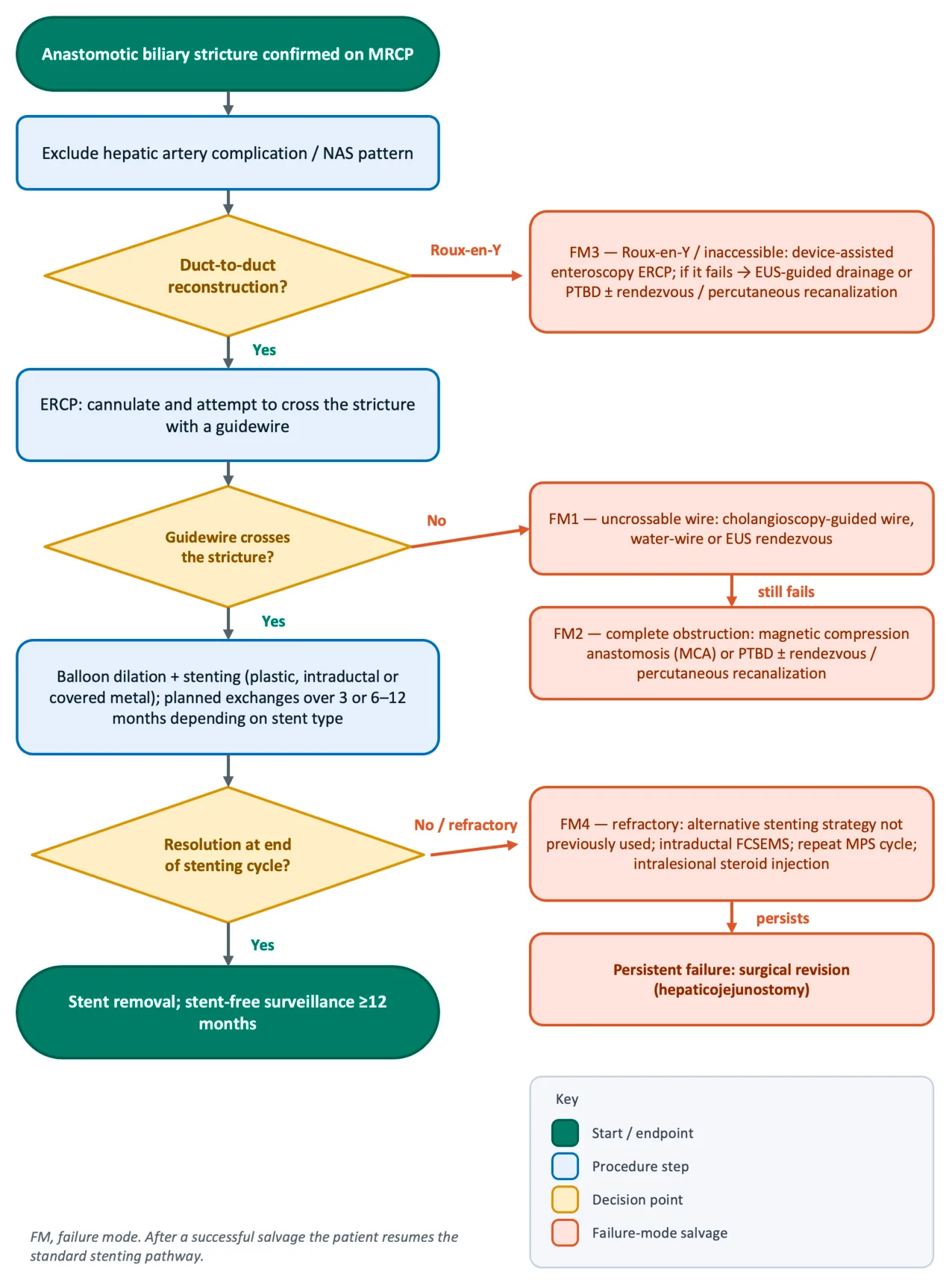
Treatment algorithm for the endoscopic management of anastomotic biliary stricture, organized around the four principal modes of endoscopic failure (FM1–FM4). FCSEMS, fully covered self-expandable metal stent; MCA, magnetic compression anastomosis; PTBD, percutaneous transhepatic biliary drainage; SEMS, self-expandable metal stent.

This narrative review is organized as a clinical pathway from pathogenesis and diagnosis through first-line therapy to an explicit framework for endoscopic failure. Rather than cataloguing devices, we map the principal modes in which endoscopy fails: an uncrossable guidewire, a completely obstructed anastomosis, an endoscopically inaccessible (Roux-en-Y) reconstruction, and a refractory stricture, each matched to a specific salvage technique (Figure 1).

## 2. Materials and Methods

This is a narrative review. We searched PubMed/MEDLINE, Scopus, and the Cochrane Central Register of Controlled Trials from database inception to June 2026 for English-language articles on the endoscopic management of anastomotic biliary stricture after liver transplantation, combining terms for liver transplantation, biliary or anastomotic stricture, endoscopic retrograde cholangiopancreatography, and biliary stenting. The reference lists of relevant articles and recent society guidelines were screened for additional sources. Randomized controlled trials, meta-analyses, and the largest or most transplant-specific cohorts were prioritized; selection was narrative and made by the authors. No formal systematic review protocol or risk-of-bias assessment was applied, and all pooled estimates cited are reproduced from published primary studies and meta-analyses.

## 3. Pathogenesis and Risk Factors

Anastomotic stricture is a localized, fibrotic failure of healing at the bilio-biliary or bilio-enteric anastomosis. Its pathogenesis can be traced as a sequence that begins with ischemic and chemical injury of the biliary epithelium and ends in transforming growth factor-β (TGF-β)–driven fibrotic remodeling of the anastomotic wall. The anatomic substrate for this process is the precarious blood supply of the terminal bile duct, which is nourished almost exclusively by the peribiliary vascular plexus arising from the hepatic artery. Transection and re-anastomosis devascularize the duct ends, so the anastomotic segment depends on collateral arterial inflow re-established across the anastomosis and is intrinsically susceptible to relative ischemia [1].

On this vulnerable substrate, several insults initiate epithelial injury. Cold preservation and warm reperfusion damage cholangiocytes and the peribiliary glands that act as their progenitor reservoir, and exposure of the injured epithelium to concentrated, hydrophobic, cytotoxic bile salts during the period of impaired bile flow compounds the damage, particularly in marginal grafts [6]. Early anastomotic bile leak, which is common after duct-to-duct reconstruction, adds a potent local inflammatory stimulus. Where cholangiocyte regeneration cannot keep pace with injury, the wound heals not by epithelial restitution but by scar formation: biliary stricture in general arises from the interplay of three processes: epithelial regeneration, inflammation, and fibrosis [7].

The final common pathway is fibrosis. Periductal fibroblasts are activated into collagen-secreting myofibroblasts, a program driven substantially by TGF-β signaling through its type I receptor (ALK5). Experimental support, albeit in a bilio-enteric (choledochojejunostomy) anastomosis rather than a duct-to-duct transplant model, comes from a study in which pharmacologic inhibition of the TGF-β type I receptor reduced anastomotic fibrosis and stricture formation [8]. The result is a focal, dense, often concentric scar confined to the anastomosis, mechanistically and morphologically distinct from the diffuse, multifocal ischemic or immune-mediated injury that defines non-anastomotic strictures. Superimposed technical and mechanical factors, including fine-caliber ducts, anastomotic tension, donor–recipient duct size mismatch, electrocautery injury, and suboptimal surgical technique, converge on this same fibrotic endpoint [1].

Reported incidence varies with anastomotic type, donor type, and follow-up duration. In deceased-donor, duct-to-duct series, the cumulative risk rises over time, reaching approximately 6.6%, 10.6%, and 12.3% at 1, 5, and 10 years, respectively [1], while some contemporary single-center cohorts report AS in up to a quarter of recipients (24.9%) [9], and stricture alone accounts for the majority of all biliary complications [10]. The most consistently identified risk factors relate to donor type and graft quality. Livers from donation after circulatory death (DCD) carry substantially higher biliary complication rates than donation-after-brain-death (DBD) grafts (47% vs. 26% in one large single-center analysis). This excess is largely ischemic and non-anastomotic, reflecting the greater ischemic burden of DCD grafts [11]. Among DCD recipients specifically, roughly half develop strictures requiring ERCP, and early post-transplant cholestatic laboratory trajectories predict risk [12]. Living-donor transplantation, with its smaller-caliber ducts and more complex, frequently multiple anastomoses, likewise carries higher rates of biliary complications than deceased-donor transplantation [13].

Donor-graft preservation also bears on biliary complications. Hypothermic oxygenated machine perfusion, particularly in DCD grafts, reduces ischemia–reperfusion injury and has shown sustained benefit for ischemic and non-anastomotic biliary complications. In the DHOPE-DCD trial (dual hypothermic oxygenated machine perfusion in donation after circulatory death), dual hypothermic oxygenated machine perfusion lowered the incidence of symptomatic non-anastomotic strictures, a benefit that persisted at long-term follow-up [14], and a meta-analysis of randomized machine-perfusion trials shows the same direction of effect [15]. This should not be extrapolated directly to isolated anastomotic stricture, which remains more dependent on local anastomotic healing, duct caliber, tension, vascularization of the duct ends, and surgical factors. Normothermic machine perfusion (NMP) permits preservation the graft at physiological temperature and is the dominant dynamic-preservation platform in North America (the OrganOx metra and the TransMedics Organ Care System). In the OrganOx randomized trial, there was no significant difference in bile-duct complications overall [16], whereas the Organ Care System trial reduced ischemic biliary complications (1.3% versus 8.5% at 6 months and 2.6% versus 9.9% at 12 months) [17]. However, as with hypothermic perfusion, the proven biliary benefit of NMP concerns ischemic, non-anastomotic injury. Observational cohort and registry analyses are concordant: NMP reduces ischemic cholangiopathy but does not reduce overall biliary complications, and its effect on anastomotic strictures specifically is unproven [18,19]. Machine perfusion is therefore best framed as a strategy to reduce the DCD-associated ischemic biliary burden of NAS rather than as a proven preventive intervention for AS.

The early immunosuppression protocol has also been scrutinized, although its specific contribution to anastomotic, as distinct from non-anastomotic, stricture remains limited and indirect. Mammalian target of rapamycin (mTOR) inhibitors delay wound healing [20], and their de novo use in the first month after transplantation is generally deferred on safety grounds [21]; calcineurin-inhibitor choice (cyclosporine versus tacrolimus) shows at most weak and inconsistent associations with biliary stricture, with host factors such as the IL-28B genotype modulating biliary complication risk in cyclosporine-treated recipients [22].

Rather than the drug class, the adequacy of immunosuppression appears to be what matters. No single regimen has been clearly identified as a direct cause of anastomotic stricture. The more consistent signal is that under-immunosuppression drives risk by permitting acute cellular rejection and the anastomotic fibrosis that follows. Indeed, acute cellular rejection is itself an independent or associated risk factor for post-transplant biliary stricture in both deceased-donor and living-donor cohorts [23,24]. Optimizing tacrolimus exposure is therefore a plausible modifiable strategy: an adequate cumulative exposure to tacrolimus (CET) with low intra-patient variability is associated with fewer biliary complications [25], and a higher time in therapeutic range (TTR) predicts better composite outcomes, including fewer biliary complications and rejection episodes [26]. These associations, however, derive from composite biliary or graft endpoints and should be read as indirect for AS. No immunosuppressive regimen has been convincingly shown to prevent AS: the cyclosporine–IL-28B interaction [22] remains hypothesis-generating, and although mTOR inhibitors have theoretical antifibrotic effects, their early de novo use raises wound-healing concerns and they should not be regarded as protective against AS outside prospective evidence [20,21]. Table 1 summarizes the principal risk factors for AS.

## 4. Diagnosis and Timing of Intervention

Because the clinical and biochemical presentation of AS is non-specific (pruritus, alterations in cholestatic liver tests, or merely an asymptomatic rise in alkaline phosphatase and bilirubin), imaging confirmation precedes intervention. Magnetic resonance cholangiopancreatography (MRCP) is the non-invasive gatekeeper. Across transplant-specific series, it shows high diagnostic accuracy, with reported sensitivity for anastomotic narrowing ranging from roughly 90% to 100% and a high negative predictive value, allowing a normal MRCP to defer invasive cholangiography [27,28,29,30,31,32,33,34]. A normal MRCP substantially lowers the probability of a clinically relevant stricture but does not exclude it, particularly early after transplantation or in fibrotic, poorly distensible ducts; persistent cholestasis, cholangitis, or a high clinical suspicion should prompt reassessment and, in selected cases, direct cholangiography. In a representative prospective comparison after living-donor transplantation, MRCP achieved 96.9% sensitivity and positive predictive value against direct cholangiography [34], and a double-blind transplant series found MRCP sensitivity approaching 100% with specificity in the 80–90% range [30]. MRCP also maps the anatomy, length, and severity of the stricture and distinguishes AS from the multifocal pattern of NAS, directly informing the choice of access route [35]. Because hepatic artery stenosis or thrombosis can cause or accompany biliary strictures and worsen prognosis, arterial patency should be confirmed (by Doppler ultrasound or CT angiography) before a stricture is attributed to a purely anastomotic mechanism; in one cohort, anastomotic strictures were far more frequent with hepatic artery stenosis than without (35% vs. 8%) [3]. In general, because cholestatic liver tests are non-specific, biliary stricture is only one of several possible causes: rejection, drug-induced injury, recurrent disease, and infection should also be considered. When imaging does not confirm a stricture, or cholestasis persists despite adequate drainage, liver biopsy (alongside the vascular assessment above) is necessary to identify these alternatives [36].

Once a stricture is confirmed, the access modality follows the reconstruction. In duct-to-duct recipients, the large majority, ERCP is simultaneously confirmatory and therapeutic and is therefore the first-line approach. Percutaneous transhepatic cholangiography (PTC) and combined biliary drainage are reserved for failed endoscopic cannulation, complex angulated strictures, or bilio-enteric (Roux-en-Y) anatomy that is endoscopically inaccessible without device-assisted enteroscopy; in mixed series, the great majority of patients are managed endoscopically, and only a minority require PTC [34,37]. A combined “rendezvous” approach is useful when a guidewire cannot be advanced antegrade across a tight stricture.

The optimal timing of intervention is less rigorously defined. AS tends to present within the first post-transplant year, with a median time to diagnosis of roughly three months and the majority appearing within six months [9], although later strictures occur and contribute to the rising cumulative incidence [1]. Non-anastomotic strictures, by contrast, tend to present later, are more often multifocal and intrahepatic, and reflect ischemic injury rather than local anastomotic healing [1,3]. When a stricture is detected before symptoms, management follows the biochemical picture: recipients with normal liver tests and no cholangitis can be monitored with close biochemical and imaging surveillance, whereas progressive cholestasis, even without jaundice, or any episode of cholangitis warrants prompt cholangiography and intervention. A structured biliary assessment around 12 months after transplantation, as proposed by recent consensus, helps standardize this surveillance [38]. Pragmatically, once a significant AS is confirmed by MRCP, prompt ERCP is favored to relieve cholestasis and limit secondary parenchymal injury; very early post-operative narrowing may partly reflect edema and can occasionally settle, but persistent or progressive strictures warrant treatment.

## 5. Balloon Dilation: An Adjunct, Not an Alternative, to Stenting

Balloon dilation is often the first mechanical maneuver in the endoscopic treatment of AS. When the stricture is too tight to admit a stent directly, a low-profile balloon is advanced over the guidewire and inflated across the fibrotic narrowing to restore a lumen and allow passage and seating of one or more stents. It is not obligatory, however; a stent (particularly a single fully covered metal stent, or a single plastic stent across a stricture that already admits it) can be placed without prior dilation. As a stand-alone therapy, dilation is durably inadequate: the remodeled scar recoils, and strictures treated by dilation alone recur in a large proportion of patients, so contemporary practice uses dilation to prepare the stricture for stenting rather than to treat it definitively [2,4,39].

The evidence that dilation must be coupled with stenting is consistent across series. A recent comparative analysis showed that balloon dilation combined with stent placement yields substantially better long-term patency than dilation alone, which in one long-term living-donor cohort recurred in 57.9% of patients versus 4% after plastic stenting [40], and is now reserved for the uncommon, very early, edematous, or hairline narrowing, or used as an adjunct at each stenting session to accommodate an increasing stent burden [41]. When performed, graded dilation (typically to 4–8 mm, bounded by the donor and recipient duct caliber and by the risk of overdilation) precedes stent insertion and is repeated at subsequent exchanges.

The principal hazard is mechanical. Aggressive dilation across a fresh anastomosis can precipitate perforation or bile leak, particularly in the early post-operative weeks when the suture line is immature. Balloon diameter and inflation pressure should therefore respect duct caliber and the age of the anastomosis. Dilation is thus enabling but not curative and is best regarded as the opening move of a stenting protocol rather than a therapy in its own right [39].

## 6. Stent Strategy: Multiple Plastic Stents Versus Fully Covered Metal Stents

The choice of stent has the strongest evidence base of any question addressed in this review, comprising five RCTs and several meta-analyses. The historical standard is sequential placement of an increasing number of side-by-side plastic stents, exchanged at intervals over 6–12 months to progressively remodel the stricture. Fully covered self-expandable metal stents (FCSEMS) were introduced to deliver an equivalent or larger luminal diameter through a single device, thereby reducing the number of ERCPs.

The randomized data, from both mixed and transplant-specific populations, are remarkably consistent on the central trade-off. In the largest trial, a multicenter RCT of treatment-naïve benign biliary strictures (N = 112, of whom 73 were post-transplant), FCSEMS were non-inferior to plastic stents for stricture resolution and required significantly fewer ERCPs to achieve it (mean 2.14 vs. 3.24; *p* < 0.001) [42]. A single-center transplant RCT comparing a 6-month FCSEMS course with plastic stents exchanged every 3 months over a year found no significant difference in resolution (83.3% vs. 96.5%; *p* = 0.19) but observed significantly more recurrences after FCSEMS (32% vs. 0%; *p* < 0.01), tempering the appeal of the metal stent’s procedural economy [43]. A multicenter transplant RCT whose primary endpoint was the number of interventions until resolution similarly favored FCSEMS for procedural burden [44], and a smaller randomized trial confirmed comparable resolution (10/10 vs. 8/10) with fewer ERCPs in the FCSEMS arm [45]. A long-term single-center RCT (n = 30) reinforced the same trade-off, with comparable radiological success (73% vs. 93%; *p* = NS) but more recurrence, re-treatment, and stent migration with FCSEMS, and overall cost parity [46]. Pooling these data, a meta-analysis of seven studies (four randomized) found resolution marginally, though not significantly, higher with metal stents (OR 1.38, 95% CI 0.60–3.15), with low heterogeneity and only a non-significant trend in recurrence [47].

The principal problem of FCSEMS is migration, which can necessitate repeated procedures. Partially covered designs reduce migration but can permit tissue ingrowth through their uncovered segments, which complicates later removal. Intraductal or anti-migration designs have been explored to mitigate this [48,49]. Dedicated intraductal designs address migration directly: a short, unflared fully covered metal stent with a long retrieval lasso, deployed entirely above the papilla, resists migration and avoids stent-induced ductal injury. In a prospective multicenter transplant series, it achieved 100% technical and 81% clinical success, with no ductal injury and only 16% early migration [49]. A purpose-built device of this type, the Kaffes stent, combines an antimigration waist with a long duodenal retrieval string: in the original transplant series, it resolved every stricture (100%), with migration in a single case (2.8%) and recurrence in 24% [50]. A later transplant cohort confirmed a low complication rate and supported longer indwell times [51]. Longer-term transplant data show that a substantial minority of patients remain stricture-free years after FCSEMS removal, but recurrence over a 5-year horizon is not negligible [52,53].

Taken together, current randomized evidence and the updated RCT-only meta-analysis support similar efficacy and safety for FCSEMS and MPS. In a 2025 meta-analysis of five RCTs (245 patients), stricture resolution, recurrence, adverse events, and cost did not differ significantly, whereas FCSEMS reduced the number of ERCP sessions (by about 1.7; *p* = 0.005) and shortened treatment duration (by about 96 days; *p* = 0.03) [54]. This procedural advantage underlies the ASGE conditional preference for covered self-expandable metal stents in extrahepatic post-transplant strictures [4]. However, this equivalence should be read cautiously: the constituent randomized trials are individually small (the largest enrolled 112 patients, several fewer than 65), and the pooled confidence intervals are wide. The result may reflect therefore an absence of a demonstrated difference rather than established equivalence. This is clearest for recurrence: covered metal stents were followed by more recurrences than plastic stents in the individual trials (32% versus 0%, *p* < 0.01, and 36% versus 7% [43,46]), and the RCT-only meta-analysis found a pooled recurrence risk ratio of 2.22 [54], a more than doubled risk that is directionally concordant with these trials. Thus, non-significance may reflect the few events across small trials rather than a true absence of effect. The choice remains therefore individualized: migration, a possible recurrence signal in selected trials, duct caliber, proximity to the hilum, living-donor anatomy, cost, and local expertise all bear on it, and MPS remain a valid standard where durability and precise ductal calibration are prioritized.

A broader comparative meta-analysis that also included intraductal SEMS found no clear difference in resolution or recurrence across stent types, but suggested that intraductal SEMS may reduce migration and may be the most cost-effective option [55]. Because it incorporated non-randomized evidence and heterogeneous devices, this analysis is best read as hypothesis-generating and complementary to the randomized FCSEMS-versus-MPS comparison. Table 2 summarizes the randomized trials and meta-analyses of stent strategy.

In our view, the migration risk of FCSEMS is not a trivial concern, and for this reason, we prefer sequential multistenting with plastic stents in most patients, reserving fully covered metal stents for the selected situations in which minimizing the number of procedures is paramount. Where available, a dedicated intraductal anti-migration stent is an attractive compromise, retaining the procedural economy of a metal stent while limiting its main drawback. As regards the multistenting technique itself, and in the absence of a prospective comparative evaluation of sequential stent addition versus complete exchange, our practice is to add a single stent at each session while leaving those already in place, and to exchange all indwelling stents only in the event of intercurrent cholangitis.

## 7. Stent-Exchange Strategy: Complete Exchange Versus Sequential Addition

In contrast to the well-studied issue of stent type, the optimal stent-exchange strategy remains to be determined. The conventional protocol, incremental dilation and stent exchange (IDSE), repeats ERCP at roughly 3-month intervals, each time removing the existing stents, dilating, and replacing them with an increasing number of plastic stents until the stricture is remodeled. Long-term series of this approach report excellent results: in one retrospective cohort treating AS with a progressively increasing number of plastic stents, resolution was achieved in 50 of 51 patients [56]. The cost of this efficacy is a long treatment course with many lengthy, radiation-intensive ERCPs.

An alternative philosophy is sequential stent addition (SSA) without routine removal, exchange, or dilation. At each session, additional stents are simply placed alongside the existing ones across the stricture. A direct retrospective comparison of IDSE with SSA reported comparable stricture resolution while SSA reduced procedure length, radiation exposure, and cost [57]. A prospective analysis of a sequential multistenting protocol without stent removal corroborated favorable clinical success and acceptable adverse-event rates [58]. Related “rapid-sequence” protocols, which compress the interval between sessions and reach a maximal stent burden quickly, achieved durable resolution with a mean of 3.4 ERCPs per patient [59]. The plastic-stent arms of the randomized stent-type trials, which exchanged stents every 3 months, anchor the conventional comparator [43].

No randomized trial has directly compared complete exchange with sequential addition. The available evidence is retrospective or single-arm prospective, heterogeneous in stent number, caliber, and interval, and susceptible to selection and center effects. Aggressive escalation of stent number, by either route, and sequential addition without exchange are plausible, resource-sparing options that warrant prospective comparative testing, with endpoints that should include the number of ERCPs, fluoroscopy time, cumulative per-patient adverse events, cost, and stent-free recurrence at 12 to 24 months.

## 8. Defining and Standardizing Failure: A Proposed Framework

Comparisons across the literature are hampered by a fundamental problem: there is no consensus on the definition of treatment success or failure. Endpoints differ across even the highest-quality studies. Definitions of success vary: cholangiographic resolution at final stent removal [43], the number of ERCPs needed to reach resolution [42,44], or a composite of cholangiographic patency with normalized liver function tests and sustained clinical patency over follow-up [45,56]. Recurrence, arguably the most patient-relevant endpoint, is variably defined and ascertained over follow-up windows ranging from one year to several, which alone can explain divergent recurrence rates between otherwise similar trials (e.g., 32% vs. 0% between arms of a single RCT over differing effective exposure) [43].

A workable, evidence-consistent operational definition emerges from synthesizing these studies: (1) technical success is the ability to traverse and stent the stricture; (2) treatment success (resolution) is cholangiographic and biochemical normalization permitting definitive stent removal at the end of a planned stenting cycle (commonly 6–12 months); (3) durable success is the absence of clinically or radiologically significant restenosis requiring re-intervention over at least 12 months of stent-free follow-up. However, we must consider two caveats attached to these definitions. First, we should require biochemical improvement rather than complete biochemical normalization whenever concurrent graft pathology (rejection, steatosis, recurrent disease, or vascular abnormalities) is suspected; we therefore anchor this definition to a return toward the patient’s own post-transplant baseline rather than to a fixed threshold. Second, the 12-month threshold is admittedly arbitrary. We adopt it not as a biological boundary but as a pragmatic, uniform cut-off, recommended to harmonize the reporting of recurrence and durability across studies. Against this, a refractory or failed stricture is one that persists despite a complete, maximally escalated stenting cycle, or that recurs early after removal, at which point FCSEMS rescue, a further plastic-stent cycle, percutaneous therapy, or surgical revision (hepaticojejunostomy) is considered. Endoscopic therapy resolves the large majority of anastomotic strictures, with resolution rates of roughly 80–95% across randomized and cohort series [43,46,54]; a minority, on the order of 5–20%, prove refractory to endoscopy by any definition and proceed to percutaneous drainage or surgical revision [47]. Standardizing these endpoints across centers is the single most valuable methodological improvement the field could adopt.

We therefore propose the pragmatic framework set out in Table 3, a harmonized set of operational definitions together with a minimum reporting dataset for future studies. It is important to note that these are author-derived provisional definition offered to seed formal, multidisciplinary validation rather than to pre-empt it. Adopting a single definition of treatment success, a stated recurrence window, and adverse-event grading according to the ESGE consensus [60] would do more to advance the field than any individual technical refinement.

A recent BileducTx consensus has proposed a location-based classification of post-transplant biliary complications, separating anastomotic complications from intrahepatic and hilar post-transplant cholangiopathy and recommending standardized reporting of biliary reconstruction, arterial complications, timing, follow-up, and clinical severity, with assessment at 12 months and MRCP or ERCP as the diagnostic reference [38]. This consensus provides a useful nomenclature for classifying post-transplant biliary disease, but it does not resolve the endoscopy-specific problem addressed here, namely how to define technical failure, treatment failure, recurrence, and durable stent-free success after AS therapy.

## 9. A Framework for Endoscopic Failure: Matching the Failure Mode to Its Salvage

Most reviews organize the endoscopic therapy of AS around the choice of device. We instead frame the difficult and refractory cases around the way endoscopy fails because the salvage technique is dictated by the mode of failure, not by the stent. In practice, endoscopic failures fall into four principal modes: (1) the guidewire cannot be advanced across the stricture; (2) the anastomosis is completely obstructed; (3) the reconstruction is endoscopically inaccessible (Roux-en-Y); and (4) the stricture is refractory despite a complete stenting cycle. Figure 1 maps the standard pathway and the salvage matched to each mode, while Table 4 summarizes the supporting evidence.

### 9.1. Failure Mode 1, the Uncrossable Guidewire

**Figure 2:**
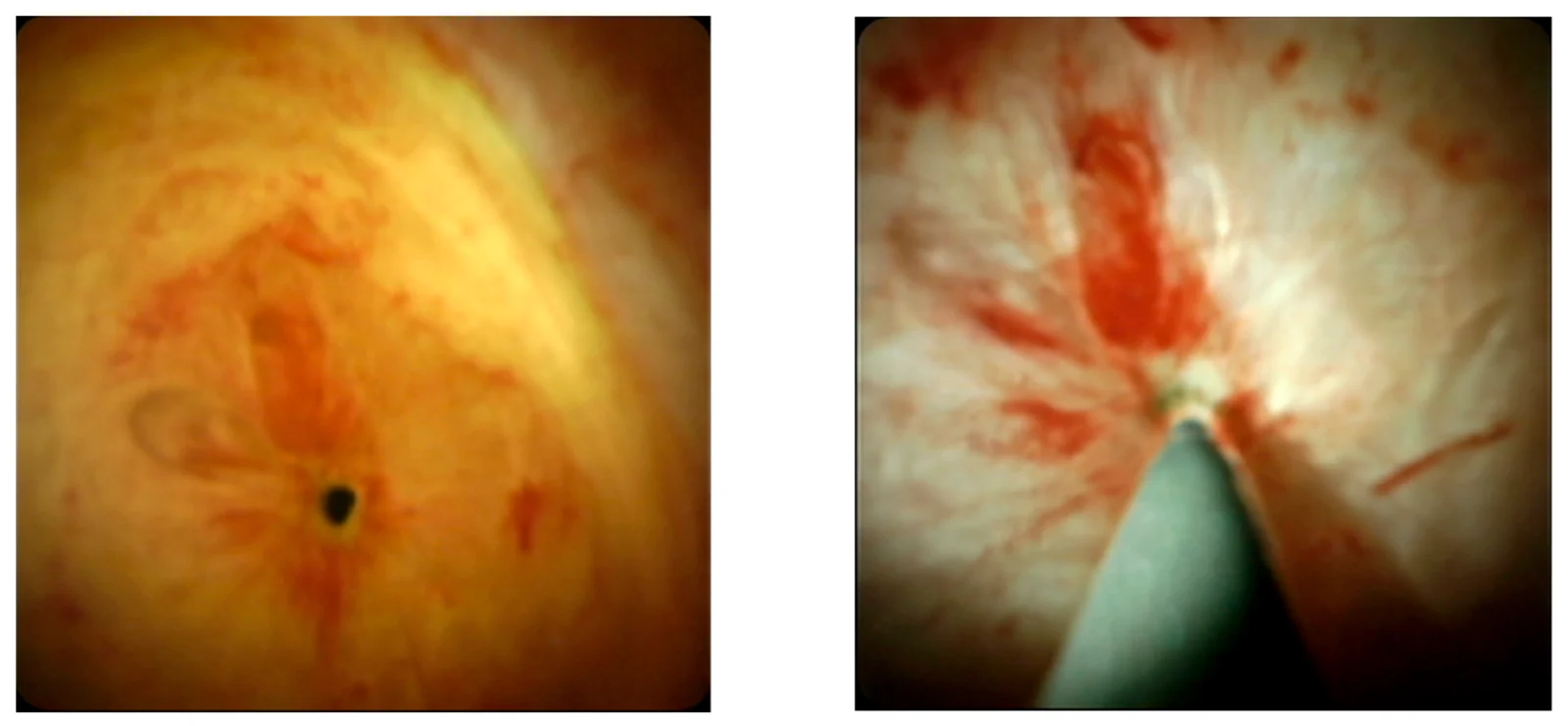
Cholangioscopic appearance of a narrow AS that was passed with a 0.035” guidewire under direct visualization.

The most common reason a standard ERCP fails is inability to cross a tight or angulated stricture with a guidewire under fluoroscopy. Digital single-operator cholangioscopy provides direct intraductal visualization, helps distinguish a focal anastomotic scar from a multifocal non-anastomotic pattern, and enables cholangioscopy-guided guidewire passage. In a prospective study of living-donor recipients with difficult AS after failed standard ERCP (SPYPASS-2), cholangioscopy-assisted access achieved technical success (guidewire placement across the stricture) in 92.5% (37/40) of these otherwise failed cases, with clinical success in 82.5%. Adverse events, though all mild, were not negligible (post-ERCP pancreatitis 15.0%, cholangitis 10.0%, bleeding 2.5%) [62] (Figure 2). A water-wire technique and an EUS-guided rendezvous are alternative ways to deliver a wire across the lesion [63]. Once the wire is across, the patient returns to the standard dilation and stenting pathway.

### 9.2. Failure Mode 2, the Completely Obstructed Anastomosis

When the anastomosis is completely obstructed and neither antegrade nor retrograde guidewire passage succeeds, magnetic compression anastomosis (MCA) may offer a non-surgical route to recanalization. The device comprises two rare-earth magnets, a smaller daughter magnet advanced to one side of the obstruction through the percutaneous transhepatic tract and a larger parent magnet brought to the other side at ERCP or through an existing drain, whose mutual attraction holds the obstructed segment under continuous compression [66]. The interposed scar undergoes gradual pressure necrosis while the two duct ends are drawn together and fuse into a new channel; an internal stent or drain is then left across the neo-anastomosis to maintain patency. Because recanalization is gradual rather than sharp, MCA may reduce the risk of uncontrolled perforation or bile leak, although this inference is based on limited and heterogeneous evidence.

The supporting evidence, although drawn mostly from benign biliary strictures broadly rather than from transplant recipients alone, is encouraging. A single-center series of completely obstructed benign strictures achieved recanalization in 35 of 39 patients, with mild cholangitis in only one case and no procedure-related mortality [64]. A 2026 systematic review and meta-analysis of MCA for benign biliary obstruction pooled a technical success rate of 91% (95% CI 84–95%), a recurrence rate of about 13%, and cholangitis in roughly 3%, with the anastomosis typically created within about ten days [68]. In transplant recipients specifically, dedicated series and a combined endoscopic–percutaneous approach report successful recanalization of otherwise untreatable complete anastomotic obstructions [65,67,75]. MCA is therefore regarded as the best rescue option, in expert centers, for a complete obstruction that has defeated every conventional attempt at guidewire passage before surgical revision is considered.

### 9.3. Failure Mode 3, the Endoscopically Inaccessible (Roux-en-Y) Reconstruction

In recipients with a bilio-enteric (Roux-en-Y) reconstruction, the hepaticojejunostomy lies beyond the reach of a duodenoscope. Modern living-donor transplantation uses duct-to-duct reconstruction whenever feasible, while a Roux-en-Y hepaticojejunostomy is selected on the basis of biliary anatomy, multiple or small ducts, the underlying disease, and surgical preference. Device-assisted (single- or double-balloon) enteroscopy-assisted ERCP reaches the anastomosis and permits dilation and stenting; compared with percutaneous transhepatic drainage for biliary-enteric anastomotic strictures, an enteroscopy-first strategy achieves treatment with fewer procedures [69,70]. When enteroscopic access also fails, EUS-guided biliary drainage, hepaticogastrostomy or an EUS-guided rendezvous can decompress the system and allow the stricture to be calibrated from above; the evidence in benign disease and surgically altered anatomy is accumulating but derives largely from predominantly non-transplant, mixed cohorts and should be read as indirect for the transplant setting [71,72,76,77].

In highly selected patients with a Roux-en-Y hepaticojejunostomy in whom repeated biliary access is anticipated and enteroscopy-assisted ERCP fails, EUS-directed transenteric ERCP through a lumen-apposing stent entero-enteric conduit is emerging as a way to obtain reusable access to the biliary limb. An early multicenter cohort reported high technical and clinical success, though the data remain limited and largely non-transplant-specific [78]. This approach should remain confined to tertiary centers with combined interventional EUS and ERCP expertise.

PTBD remains a valid alternative when advanced endoscopic access is unavailable or unsuccessful: it provides immediate decompression and allows the stricture to be dilated over staged sessions, at the cost of a more invasive procedure and a temporary external or internal-external drain, and it can also serve as the percutaneous arm of a combined rendezvous with endoscopy [69,70].

### 9.4. Failure Mode 4, the Refractory Stricture

A stricture that persists despite a complete, maximally escalated stenting cycle is defined as refractory; here the options shift from access to remodeling. Intraductal fully covered metal stents, deployed entirely above the papilla and retrieved by a suture, preserve the large caliber of a metal stent while reducing migration and duodeno-biliary reflux. Comparative and prospective transplant data show resolution comparable to multiple plastic stents with fewer procedures, although their use is limited by availability, a learning curve, the risk of side-branch occlusion, anatomic suitability, and the need for retrieval and follow-up [49,51,73]. Long-term data with a modified non-flared FCSEMS further support this concept in refractory AS, with high clinical success, shorter stenting duration, and lower recurrence than plastic stents in a non-randomized comparison, and no de novo strictures [79]. These findings are best read as supportive rather than definitive because much of the observed benefit probably reflects device design and selective use in expert centers. Cholangioscopy-guided intralesional steroid injection has been used as an adjunct in refractory disease, but the evidence is limited to small series and case reports without controlled data, so it remains investigational [74]. Strictures that defeat all of these attempts are referred for surgical revision (hepaticojejunostomy).

## 10. Procedure-Related Adverse Events and Safety

The procedural burden of treating AS, which commonly requires several ERCPs over months, makes the safety of repeated endoscopy a first-order consideration, on par with efficacy. The principal adverse events of ERCP in this setting are post-ERCP pancreatitis, cholangitis, post-sphincterotomy bleeding, and perforation, together with stent-specific events (migration and occlusion). Transplant recipients are not the average ERCP population, and a small but specific literature now characterizes their risk: large database and cohort analyses indicate that ERCP is performed safely in liver transplant recipients, with an overall adverse-event profile comparable to, and for pancreatitis frequently lower than, non-transplant patients [80,81] (Table 5).

Post-ERCP pancreatitis is the most studied event. Several transplant-specific analyses report post-ERCP pancreatitis rates at or below those of the general ERCP population, possibly reflecting frequent prior sphincterotomy, biliary rather than pancreatic therapeutic intent, and the anti-inflammatory effect of maintenance immunosuppression [82,83]. This lower baseline does not abolish risk, and standard prophylaxis (rectal non-steroidal anti-inflammatory drugs, guidewire-assisted cannulation, and pancreatic-duct stenting where indicated) still applies.

Cholangitis is the other clinically important event, mostly driven by stent occlusion and incomplete drainage across a tight stricture. In cohorts undergoing repeated stenting for post-transplant strictures, post-ERCP cholangitis is among the commonest causes of unplanned readmission; adequate drainage at every session and timely stent exchange are therefore essential [81,84]. Antibiotic prophylaxis is one of the established ACG/ASGE quality indicators for ERCP and is most strongly supported in three settings: anticipated or actual incomplete relief of biliary obstruction (for example, primary sclerosing cholangitis), cholangioscopy, and orthotopic liver transplantation [85]. These two positions are complementary rather than contradictory: the quality indicator treats liver transplantation as a category in which prophylaxis is reasonable, mainly because chronic immunosuppression raises the baseline risk of infection, whereas the transplant-specific clinical guidance calibrates that indication to the measured likelihood of post-ERCP infection. Accordingly, the recommendation for transplant recipients has narrowed: because post-ERCP infection rates proved low [86], the ASGE moved from universal prophylaxis for all transplant ERCP to a conditional one, reserving antibiotics for anticipated incomplete drainage, such as ischemic cholangiopathy, multiple intrahepatic strictures, or contrast injection without stent placement [4]. Where prophylaxis is given, a single periprocedural dose appears sufficient: in transplant recipients undergoing routine ERCP, single-dose prophylaxis with ampicillin-sulbactam (a single 3 g dose) did not increase post-ERCP cholangitis compared with a multiday course (22.2% vs. 18.0%), even with dilation. This single-center observation supports, but does not establish, the safety of shorter regimens [87]. A systematic review and meta-analysis of antibiotic prophylaxis in transplant recipients undergoing ERCP found that post-ERCP infections and antimicrobial resistance remain clinically relevant despite widespread prophylaxis, which argues for prophylaxis targeted to anticipated incomplete drainage, cholangioscopy, or high-risk anatomy and, when repeated instrumentation or prior cholangitis is present, informed by previous bile cultures and local resistance patterns [88]. The more honest safety metric for these patients is the cumulative, per-patient risk of a multi-session protocol rather than the per-procedure risk, and that cumulative burden is itself an argument for procedure-sparing strategies, such as FCSEMS or sequential stent addition, where they are appropriate.

Cross-study comparison is again hampered by inconsistent reporting; adopting the European Society of Gastrointestinal Endoscopy consensus definitions and severity grading for ERCP-related adverse events would make safety data poolable across centers [60]. Given that the choice between stent strategies is, in large part, a trade-off between durability and cumulative procedural harm, safety in this field is best reported as a co-primary outcome alongside stricture resolution rather than relegated to a secondary mention.

## 11. Impact of Endoscopic Treatment on Liver-Related Outcomes

Recurrent AS after apparently successful endotherapy deserves separate reporting. In a recent single-center cohort, recurrent anastomotic biliary stricture occurred in 22.7% of patients after successful completion of an initial endoscopic course, at a median of 17 months from resolution. Repeat endotherapy, most often with covered metal stents, achieved treatment success in about 80% of recurrent cases, with adverse events in 7.4% [61]. These data support a second endoscopic course before surgical revision in suitable patients and argue for recurrence assessment that extends beyond the first stent-free year.

The ultimate justification for aggressive endoscopic therapy is preservation of the graft and the patient. Here, the evidence is observational but directionally consistent. Durable endoscopic success is associated with improved survival: in a long-term outcome analysis, achievement of long-term treatment success (≥12 months) was significantly associated with patient survival in anastomotic strictures (*p* = 0.036) [37]. Conversely, biliary complications as a whole, and particularly the combination of leak and stricture, reduce 1-, 3-, and 5-year patient and graft survival relative to recipients without biliary complications [10]. In a duct-to-duct cohort, first-line endoscopic management succeeded in roughly 80% of strictures, and outcomes were shaped more by recipient acuity at transplant than by the stricture itself once treated [9]. Historical series likewise indicate that, when AS is successfully relieved, graft and patient survival approach those of recipients without strictures, whereas DCD grafts, in which strictures cluster, carry lower baseline survival [1,11].

The reasonable interpretation is that successful, timely endoscopic resolution of AS restores bile flow and protects the graft, rendering the long-term prognosis of treated AS favorable and, in observational series, broadly comparable to that of stricture-free recipients, although this association is confounded by donor, recipient, vascular, and non-anastomotic risk. Persistent or failed strictures, along with the presence of concomitant leak or non-anastomotic disease, mark a worse trajectory. In quantitative terms, 10-year graft survival was essentially identical in recipients with and without biliary complications (a composite endpoint broader than isolated AS) in a large living-donor series (66% vs. 67%) [89]; conversely, early-onset AS, despite higher stricture resolution than late-onset disease (94% vs. 68%), carried significantly worse graft survival (*p* = 0.0001) [90], underscoring the confounding role of stricture timing. Because these data are retrospective and susceptible to confounding by indication and by donor/recipient risk, the prognostic benefit of endoscopic success should be regarded as associative rather than proven causal.

## 12. Conclusions and Research Gaps

For anastomotic biliary stricture after liver transplantation with duct-to-duct reconstruction, endoscopic therapy is established first-line treatment, achieving durable resolution in the large majority of patients and associated, in observational data, with graft and patient survival approaching that of stricture-free recipients. The evidence is strongest for the choice of stent, and shows substantive equipoise between multiple plastic stents and fully covered metal stents: comparable resolution, fewer procedures with FCSEMS, greater durability and lower migration risk with MPS. The level of evidence is lower when it comes to the management of endoscopic failure, which can be defined as an uncrossable wire, a complete obstruction, an inaccessible anastomosis, or a refractory stricture, each with a salvage technique matched to it (Figure 1).

Four gaps stand out. First, no randomized trial compares complete stent exchange with sequential stent addition, despite the latter’s promise of fewer, shorter, lower-cost, radiation-sparing procedures. Second, the absence of a consensus definition of treatment success, failure, and recurrence undermines every cross-study comparison and should be remedied by a standardized core outcome set, toward which we offer a pragmatic framework of definitions and minimum reporting items (Table 3) as a starting point. Third, the prognostic link between endoscopic success and survival rests entirely on retrospective data; prospective, risk-adjusted cohorts, ideally registry-based, are needed to establish whether, and how much, durable stricture resolution causally improves graft and patient survival. Fourth, the efficacy of periprocedural antibiotic prophylaxis in transplant recipients rests on retrospective, non-randomized data; prospective, ideally randomized, trials are needed to define who benefits and for how long, balancing infection prevention against antimicrobial stewardship. Addressing these gaps would convert a largely experience-driven practice into an evidence-based one.

## Figures and Tables

**Table 1:** Risk factors for anastomotic biliary stricture after liver transplantation.

Risk Factor	Reported Association or Effect Estimate	Source
DCD donor (vs. DBD)	Higher biliary complications (47% vs. 26%); ~50% of DCD recipients develop strictures requiring ERCP	Foley et al., 2011 [11]; Kohli et al., 2019 [12]
Living-donor graft (small or multiple ducts)	Higher biliary complication rate than deceased-donor	Gómez et al., 2009 [13]
Older donor age (>47.5 y)	Independent risk factor (OR 2.05)	Karakoyun et al., 2021 [24]
Prolonged cold ischemia time	Independent risk factor (OR 1.01 per minute)	Chok et al., 2011 [23]
Early anastomotic bile leak	Independent risk factor (OR 3.94)	Karakoyun et al., 2021 [24]
Acute cellular rejection	Independent or associated risk factor (OR 3.05)	Chok et al., 2011 [23]; Karakoyun et al., 2021 [24]
Immunosuppression (tacrolimus exposure and variability)	No regimen prevents AS; low or highly variable tacrolimus exposure is associated with more biliary complications, and higher time-in-therapeutic-range with fewer	Pan et al., 2024 [25]; Song et al., 2023 [26]
Hepatic artery stenosis	Strongly associated (anastomotic strictures 35% vs. 8%)	Dacha et al., 2011 [3]
Technical and anatomic factors (fine-caliber duct, anastomotic tension, donor–recipient size mismatch)	Recognized mechanistic contributors	Verdonk et al., 2006 [1]

AS, anastomotic stricture; DCD, donation after circulatory death; ERCP, endoscopic retrograde cholangiopancratography; OR, Odds ratio.

**Table 2:** Randomized controlled trials and meta-analyses comparing fully covered self-expandable metal stents (FCSEMS) with multiple plastic stents (MPS) for anastomotic biliary stricture.

Study (Year); Design, N	Resolution (FCSEMS vs. MPS)	ERCPs	Recurrence/Migration	Key Finding
Kaffes et al., 2014 [45]; RCT, n = 20	10/10 vs. 8/10	Fewer with FCSEMS	Migration with FCSEMS	Comparable resolution, fewer procedures
Coté et al., 2016 [42]; multicenter RCT, N = 112 (73 post-LT)	Non-inferior	2.14 vs. 3.24 (*p* < 0.001)	NR	Fewer ERCPs with FCSEMS
Tal et al., 2017 [44]; multicenter RCT	Comparable	Fewer with FCSEMS (primary endpoint)	NR	Procedural burden favors FCSEMS
Martins et al., 2018 [43]; single-center RCT	83.3% vs. 96.5% (*p* = 0.19)	Fewer with FCSEMS	Recurrence 32% vs. 0% (*p* < 0.01)	More recurrence after FCSEMS
Cantù et al., 2021 [46]; RCT, n = 30	73% vs. 93% (NS)	NR	Recurrence 36% vs. 7%; migration 29% vs. 2.6% (*p* < 0.01)	Cost parity overall; more recurrence and migration with FCSEMS
Facciorusso et al., 2018 [47]; meta-analysis (7 studies, 4 RCTs)	OR 1.38 (95% CI 0.60–3.15)	Fewer with FCSEMS	Non-significant recurrence trend	Equivalent resolution, low heterogeneity
Baraldo et al., 2025 [54]; updated SR/MA of RCTs	Comparable	Fewer with FCSEMS	RR 2.22 for recurrence (NS); migration the main FCSEMS liability	5 RCTs, 245 patients; −1.7 ERCP sessions, −96 days; equipoise confirmed

ERCP, endoscopic retrograde cholangiopancreatography; FCSEMS, fully-covered metal stents; MPS, multiple plastic stents; N, total number of patients; n, number of patients in a subgroup; NR, not reported.

**Table 3:** Proposed pragmatic framework of outcome definitions and a minimum reporting dataset for studies of endoscopic therapy of anastomotic stricture. Offered as a starting point to improve cross-study comparability and to seed a future multidisciplinary consensus; not a validated standard.

Item	Proposed Operational Definition or Reporting Requirement
**Outcome definitions**	
Technical success	Successful guidewire traversal and stent placement across the stricture at the index ERCP
Treatment success (resolution)	Cholangiographic resolution with normalization or, if graft pathology precludes it, substantial improvement of cholestatic markers (ALP, GGT, bilirubin) toward baseline, allowing definitive stent removal (typically 6–12 months)
Durable success	No clinically or radiologically significant restenosis requiring re-intervention during ≥12 months of stent-free follow-up
Refractory/failed	Persistence of stricture despite a complete, maximally escalated stenting cycle, or early recurrence after stent removal
Recurrence	Restenosis after a documented durable success; the ascertainment window must be stated explicitly
**Minimum reporting items**	
Donor and anastomosis type	DBD/DCD/living-donor; duct-to-duct versus Roux-en-Y
Stricture characteristics	Length, diameter, angulation; interval from transplant to diagnosis
Intervention detail	Balloon diameter; stent type, number, caliber, and exchange interval
Follow-up	Duration of stent-free follow-up and the defined recurrence window
Adverse events	Reported and graded per the ESGE consensus (Dumonceau et al., 2020 [60])
Stricture location	Distance of the stenosis from the hilum and from the papilla, to separate a true anastomotic stricture from a hilar or intrahepatic one
Concomitant findings	Coexisting bile leak, stones or casts, or non-anastomotic stricture features, each of which alters prognosis and management
BileducTx category	Anastomotic stricture versus hilar or intrahepatic post-transplant cholangiopathy; report biliary reconstruction, arterial complications, timing, and the highest-grade biliary complication at 12 months [38]
Late recurrence	Recurrence occurring more than 12 months after stent removal; report separately from early recurrence [61]

ALP, alkaline phosphatase; DBD, donation after brain death donor; DCD, donation after cardiac death donor; ERCP, endoscopic retrograde colangiopancreatography; ESGE, European society of gastrointestinal endoscopy.

**Table 4:** Endoscopic failure modes in anastomotic stricture and the salvage technique matched to each.

Failure Mode	Salvage Technique	Evidence Type	Representative Studies
FM1, uncrossable guidewire	Cholangioscopy-guided wire; water-wire; EUS rendezvous	Prospective + series	Cho et al., 2025 [62]; Füldner et al., 2021 [63]
FM2, complete obstruction	Magnetic compression anastomosis (MCA)	Series + SR/MA (mostly benign)	Jang et al., 2017, 2020 [64,65]; Li et al., 2020 [66]; Ünal et al., 2025 [67]; Desouky et al., 2026 [68]
FM3, inaccessible (Roux-en-Y) anatomy	Device-assisted enteroscopy ERCP; EUS-guided drainage	Comparative vs. PTBD; series (mixed)	Hammad et al., 2019 [69]; Tsujino et al., 2017 [70]; Caillol et al., 2024 [71]; Koutlas et al., 2024 [72]
FM4, refractory after full cycle	Intraductal FCSEMS; cholangioscopy-guided steroid	Comparative + prospective; early	Sissingh et al., 2023 [73]; Lim et al., 2024 [51]; Yoo et al., 2020 [49]; Franzini et al., 2019 [74]

ERCP, endoscopic retrograde cholangiopancreatography; EUS, endoscopic ultrasound; FCSEMS, fully-covered metal stents; FM, failure mode; PTBD, percutaneous transhepatic biliary drainage; SR/MA, systematic review and meta-analysis.

**Table 5:** Procedure-related adverse events of ERCP in liver transplant recipients (transplant-specific and general ERCP safety evidence).

Adverse Event	Observation in Liver-Transplant Recipients	Source
Overall adverse-event profile	ERCP performed safely; adjusted odds of pancreatitis, bleeding, cholangitis, and sepsis comparable to non-transplant patients (Tarar); 30-day procedure-related readmission 3.3% (45/1369), no mortality (Gu)	Tarar et al., 2023 [80]; Gu et al., 2024 [81]
Post-ERCP pancreatitis	About 2–3% of ERCPs (23 of 1125, Ghambari; 22 of 730, Law), at or below general-population rates; maintenance prednisone independently protective (adjusted OR 0.22) (Law)	Law et al., 2013 [82]; Ghambari et al., 2024 [83]
Cholangitis	Post-ERCP cholangitis in 15.9% of procedures (148 of 930), mostly mild with no cholangitis-related mortality but linked to worse survival (median 9 vs. 15 years) and to non-anastomotic strictures (independent risk factor, OR 3.1) (Zhang); commonest cause of procedure-related readmission (2.4% per ERCP; Gu); driven by stent occlusion and incomplete drainage	Zhang et al., 2025 [84]; Gu et al., 2024 [81]
Bleeding/perforation	Mainly post-sphincterotomy or dilation-related; post-sphincterotomy bleeding 0.2–0.8% in transplant cohorts (Gu; Tarar); perforation rare	Tarar et al., 2023 [80]; Gu et al., 2024 [81]; Dumonceau et al., 2020 [60]
Stent migration/occlusion	Migration is the principal FCSEMS-specific event (10% with covered metal stents in Martins, up to 29% vs. 2.6% with plastic stents in Cantù); occlusion is uncommon and mainly a plastic-stent issue (1.4% in Martins), and drives stent exchange and cholangitis	Cantù et al., 2021 [46]; Martins et al., 2018 [43]

ERCP, endoscopic retrograde cholangiopancreatography; FCSEMS, fully covered metal stents.

## Data Availability

No new data were created or analyzed in this study. Data sharing is not applicable to this article.

## Abbreviations

The following abbreviations are used in this manuscript:

ACG	American College of Gastroenterology
ALK5	activin receptor-like kinase 5 (TGF-β type I receptor)
AS	anastomotic biliary stricture
ASGE	American Society for Gastrointestinal Endoscopy
CI	confidence interval
CT	computed tomography
DBD	donation after brain death
DCD	donation after circulatory death
DHOPE	dual hypothermic oxygenated machine perfusion
ERCP	endoscopic retrograde cholangiopancreatography
ESGE	European Society of Gastrointestinal Endoscopy
EUS	endoscopic ultrasound
FCSEMS	fully covered self-expandable metal stent
IDSE	incremental dilation and stent exchange
MCA	magnetic compression anastomosis
MPS	multiple plastic stents
MRCP	magnetic resonance cholangiopancreatography
mTOR	mammalian target of rapamycin
NAS	non-anastomotic stricture
NR	not reported
NS	not significant
OR	odds ratio
PTBD	percutaneous transhepatic biliary drainage
PTC	percutaneous transhepatic cholangiography
RCT	randomized controlled trial
SEMS	self-expandable metal stent
TGF-β	transforming growth factor-β
